# “I Think It’s Too Early to Know”: Gender Identity Labels and Gender Expression of Young Children With Nonbinary or Binary Transgender Parents

**DOI:** 10.3389/fpsyg.2022.916088

**Published:** 2022-08-17

**Authors:** Rachel G. Riskind, Samantha L. Tornello

**Affiliations:** ^1^Department of Psychology, Guilford College, Greensboro, NC, United States; ^2^Department of Human Development and Family Studies, Pennsylvania State University, University Park, PA, United States

**Keywords:** transgender parents, nonbinary parents, child gender expression, gender identity labels, transfamily theory

## Abstract

Little is known about gender expression among children with transgender parents. In the United States, we surveyed 64 nonbinary or binary transgender parents of children aged 18 to 71 months. Most parents reported a marginalized sexual identity and a White racial identity. Many declined to label their child’s gender identity, and this was particularly true among those with younger children. Scores indicated that, on average, children’s play was conventionally gendered. However, scores indicated significantly more gender-expansive play in the present sample than in normed samples, particularly among children assigned male at birth. Findings support transfamily theory (McGuire et al., 2016) and illustrate differences among families with nonbinary and binary transgender parents.

## Introduction

Parenthood is common among nonbinary^1^ and binary^2^ transgender^3^ people: current estimates suggest that 19–50% of transgender adults (Grant et al., 2011; Stotzer et al., 2014; Carone et al., 2021) and roughly 25% of nonbinary adults (Carone et al., 2021) are parents. Moreover, many childless transgender people are interested in becoming parents in the future (Tornello and Bos, 2017; Pang et al., 2020; Tasker and Gato, 2020; Guss et al., 2021). Social scientists, however, have only rarely studied children with transgender parents (Imrie et al., 2020; Pfeffer and Jones, 2020).

What do we know about gender among young children with nonbinary or binary transgender parents? Transfamily theory (McGuire et al., 2016) suggests that a transgender person’s presence in a family can challenge gender-related assumptions, intentionally or unintentionally, in ways that influence family processes. For example, children (6–8 years) with a transgender sibling may harbor less stereotypical gender-based beliefs (Olson and Enright, 2018); they may accept others’ gender non-conformity more than children with a cisgender sibling (Olson and Enright, 2018). The present study (a) investigates how and whether nonbinary and binary transgender parents label their children’s gender identity^4^, focusing on variation among these families, and (b) describes variation in gender conformity among children with nonbinary and binary transgender parents.

### Labeling Child Gender Identity

Gender identity self-categorization typically develops in early childhood. Many infants first verbalize their gender identity, such as “I am a boy,” or “I am a girl,” as early as 18–24 months (Zosuls et al., 2009), and many young children seem to develop a gender identity by about 3 years (Fausto-Sterling, 2021).^5^ In a rare study of transgender and cisgender children ages 3–5 years, groups were equally likely to self-categorize their gender identity (Fast and Olson, 2018). Riggs (2019) summarizes this phenomenon: “…what very young children are doing is creating groupings so as to order their world, groupings that typically have very little to do with visual inspection of the genitalia of others. That transgender children make such groupings is entirely unsurprising…” (p. 38). Riggs adds that binary transgender children use varied strategies to address misconceptions about their gender identity.

Before self-categorization, however, most legal, medical, and social systems in industrialized democracies assume fetus’ and newborns’ gender identity labels based on external genital appearance (Blackless et al., 2000).^6^ In these contexts, parents who decline to label their infants as a girl or boy remain rare enough to attract media coverage (e.g., Compton, 2018). If empirical studies about these parents exist, they have yet to be published^7^.

Unlike many cisgender parents, transgender parents may be particularly aware that misgendering young children can harm children’s mental and physical health (Ansara and Hegarty, 2011; McLemore, 2018). Nonbinary parents may be particularly attentive to the harms of pressure to adopt a binary gender identity (Richards et al., 2016). As a result, transfamily theory suggests that nonbinary and binary transgender parents are more likely than cisgender parents to wait until children can share their own self-categorization before labeling their young child’s gender identity^8^.

### Child Gender Expression

In support of transfamily theory (McGuire et al., 2016), previous research suggests that the presence of a transgender parent in a family may influence child gender expression^9^ (Langlois and Downs, 1980; Bem, 1981; Witt, 1997; Lee and Troop-Gordon, 2011; Sumontha et al., 2017; Brown and Stone, 2018). Negative experiences with childhood pressures toward gender conformity might make nonbinary and binary transgender parents less likely than cisgender parents to model or encourage children to conform to gendered expectations. Parents’ gender ideologies seem to influence child gender expression (but not identity^10^). Peers, media, teachers, parents, and other family members can influence child gender expression by directly and indirectly communicating gendered norms and attitudes to children (Langlois and Downs, 1980; Bem, 1981; Witt, 1997; Lee and Troop-Gordon, 2011; Brown and Stone, 2018). For example, parents with more flexible gender role ideology and less gender-typed division of unpaid labor report that their children’s behavior conforms less to gendered expectations (Sumontha et al., 2017).

There is little previous research on gender expression among children with transgender parents. Transfamily theory (McGuire et al., 2016) and research on infants and young children with lesbian, gay, or heterosexual parents suggest that parent and child gender conformity would be positively associated (Bruun and Farr, 2020). With some exceptions (Rahilly, 2015), transgender parents tend accept their children’s gender non-conforming attitudes and expression (Ryan, 2009).

#### Parent Sexual Orientation and Child Gender Expression

Many transgender parents also have marginalized sexual identities (Pfeffer and Jones, 2020), and some cisgender parents with marginalized sexual orientations are non-conforming in their gender expression. Given the lack of previous research on gender expression of children with transgender parents, parent sexual orientation studies may help inform hypotheses about transgender parents.

Some evidence suggests that child gender expression is similar across parent sexual orientations. Among children, gendered play-based activities (such as play with dolls or trucks) are salient components of gender expression, and play-based interests (such as princesses or soldiers) reflect children’s gender roles, ideologies, and expectations (Davis and Hines, 2020). Children with same-sex parents displayed typical gender development in two studies of childhood gender-typed play behavior: one from a representative United Kingdom sample (Golombok et al., 2003) and one from a longitudinal U.S. study about families formed through adoption (Farr et al., 2018). The U.S. research group studied gender-typed play behavior in infancy and early childhood and children’s clothing in middle childhood (Farr et al., 2018; Bruun and Farr, 2020), which were similar across parental sexual orientation.

However, findings in this area are mixed. In some studies of families formed through adoption, children with lesbian parents are less gender-conforming than those with heterosexual or gay parents (Goldberg et al., 2012; Goldberg and Garcia, 2016; Bruun and Farr, 2020). One study included clothing observations in infancy and early childhood (Bruun and Farr, 2020), and others relied on parent-reported early childhood gender-typed play behavior (Goldberg et al., 2012; Goldberg and Garcia, 2016). This variation in findings might be attributed to differences in child age, the measure of child gender expression, or other methodological choices that varied between studies.

Why might some studies find that children with lesbian mothers are less gender-conforming than those with heterosexual parents or gay fathers? LGBTQ parents, particularly those whose social locations afford them the power to resist pressures to conform, may be more likely than other parents to offer their children a “gender buffet” of gender expression options (Averett, 2016). For example, parents’ decisions about which gender-typed toys are present in the home may affect children’s gendered toy preferences (Boe and Woods, 2018). Such socialization can include encouraging or allowing their children to engage with gender-expansive and gender-conforming toys, clothing, and activities (Averett, 2016). However, this finding might not generalize to transgender adults, who are more likely than cisgender lesbian, gay, or bisexual peers to conceptualize gender identity as mostly innate (Catalpa et al., 2019). Researchers have yet to address whether and how belief in the innateness of gender identity may influence child socialization.

### Nonbinary and Binary Transgender Parents

A growing body of research notes differences between the experiences of nonbinary and binary transgender adults (e.g., Factor and Rothblum, 2017; Bradford and Catalpa, 2019; Catalpa et al., 2019). For example, Catalpa et al. (2019) found that genderqueer adults reported more binary-challenging gender expression and more change over time in gender identity, expression, or both than transgender binary participants. Similarly, genderqueer participants in a large, national U.S. study described themselves as lower in feminine personality traits and higher in masculine ones than did transgender women (Factor and Rothblum, 2017). In a more racially diverse sample, nonbinary adults were more likely to reject the idea that gender is “fixed, strong, and consistent” than were their binary transgender and cisgender counterparts (Bradford and Catalpa, 2019, p. 71).

These differences suggest that nonbinary parents may be more likely than binary transgender parents to model gender-fluid or binary-challenging behaviors and interests and to resist pressures to assign their child a binary gender identity label. Few studies, however, have analyzed possible differences between nonbinary and binary transgender parents (but see Tornello et al., 2019). There is some evidence for age/cohort differences between nonbinary and binary transgender parents: in a large U.S. sample, nonbinary parents were younger—and more likely to become parents after a gender transition–than binary transgender parents (Tornello et al., 2019). No previous studies have described gender expression or gender identity labels among children with nonbinary parents (Brown and Rogers, 2020).

### The Current Study

Although transgender people and their families are more familiar to the cisgender public than in years past (Pfeffer and Jones, 2020), few studies have focused on children with transgender parents. Further, despite recent evidence that nonbinary adults may be even more likely than binary transgender adults to be parents (Carone et al., 2021), no studies have described subsamples of children with nonbinary parents. The current study addressed four research questions.

#### Question 1: How Many Transgender and Nonbinary Parents Labeled Their Child’s Gender Identity?

Transfamily theory suggests that transgender people challenge common gendered assumptions in ways that affect family processes (McGuire et al., 2016). Accordingly, we hypothesized that (H1) many nonbinary and binary transgender parents would decline to label their young child’s gender identity.

#### Question 2: How Did Parents Describe Their Children’s Gendered Play?

We described gender expression scores for children in the present sample, adjusting for child age and sex assigned at birth, and compared scores for the present sample to normed samples. Following most research on this gender expression measure among children with lesbian and gay parents (Goldberg et al., 2012; Goldberg and Garcia, 2016; Bruun and Farr, 2020), we hypothesized that (H2) nonbinary and binary transgender parents would describe their child’s gender expression as typical or slightly expansive (non-conforming).

#### Question 3: Which Transgender and Nonbinary Parents Labeled Their Child’s Gender Identity?

We were interested in investigating how child gender development might predict whether or not parents label their child’s gender identity. Children’s abilities to understand and verbalize their gender identities develop rapidly during late infancy and early childhood (Fausto-Sterling, 2021), so we investigated child age as a relevant predictor. Nonbinary adults conceptualize gender development differently than binary transgender adults (Catalpa et al., 2019), so we investigated whether parent gender identity would predict labeling.

We hypothesized that (H3) the following parents would be most likely to label their child’s gender identity: binary transgender parents (as opposed to nonbinary parents), parents with older children (as opposed to parents with younger children), and parents who described their child’s gender expression as more gender-conforming (as opposed to those who described their child’s gender expression as gender-expansive). We also explored the roles of child sex assigned at birth and family socioeconomic status (SES).

#### Question 4: Which Transgender and Nonbinary Parents Described Their Children as Most Gender-Expansive?

Based on Catalpa and colleagues’ findings that nonbinary adults conceptualize gender differently from binary transgender adults, we hypothesized that (H4) nonbinary parents would describe their children as more gender-expansive than would binary transgender parents. Although we adjusted according to child age and sex assigned at birth, we explored each variable’s role, as they are associated with gender expression in normed samples^11^ (Golombok and Rust, 1993; Golombok et al., 2008). We also explored parent SES and timing of parent gender transition relative to child age; the latter has been associated with psychological outcomes among children with transgender parents (White and Ettner, 2007).

## Materials and Methods

### Participants

Participants were 64 nonbinary or binary transgender parents with at least one child aged 18–71 months, a subsample of the Gender Diverse Parents Study (Tornello et al., 2019). To be eligible to participate, participants had to be at least 18 years old, reside in the United States, have at least one child, and describe their gender identity as different from their sex assigned at birth. To maintain data point independence, only one person from each couple was eligible. Since the current study focused on early childhood, we excluded from the subsample participants whose eldest child was 72 months or older (*n* = 119), under 18 months old (*n* = 6), or whose child’s age was not noted (*n* = 10).

Participants and their children were predominantly White (84 and 82%, respectively), few identified as members of a racially marginalized group (16 and 18%, respectively; see Table 1 for all demographic details). However, their sexual identities, gender identities, SES, and geography were diverse. Just over half of participants (57%) and their partners (58%) held bachelor’s degrees or higher. The median annual household income was USD 61,000, on the border between lower-middle- and middle-class annual incomes (Kearney et al., 2013), with a quarter of participants below USD 40,000 and another quarter over USD 87,500. Participants reported varied gender and sexual identity labels, and 94% reported a marginalized sexual identity (in addition to a marginalized gender identity). Most participants (84%) reported a current committed romantic relationship; these relationships had, at time of data collection, lasted an average of almost a decade (*M* = 9.63 years, 95% CI [8.51, 10.74]).

**TABLE 1:** Demographic characteristics of nonbinary and binary transgender parent participants, their children, and their partners.

	Participants *n* = 64	Children *n* = 64	Partners *n* = 57
	*M* *[95% CI]*	*M* *[95% CI]*	*M* *[95% CI]*
Age (years)	33.61 [32.34, 34.87]	4.06 [3.76, 4.37]	35.33 [34.17, 36.48]
	%	%	%
**Gender identity**			
Nonbinary*^a^*	56	0	20
Transgender man or boy	22	0	2
Transgender woman or girl	20	2	4
Multiple identities	2	0	0
No label*^b^*	0	41	0
Cisgender man or boy	0	33	21
Cisgender woman or girl	0	25	52
Cisgender (did not specify further)	0	0	2
**Sexual identity**			
Queer	42	–	28
Pansexual	16	–	9
Bisexual	13	–	19
Lesbian	8	–	9
Heterosexual	6	–	26
Demisexual	5	–	4
Asexual	3	–	2
Choose not to label	3	–	2
Additional sexual identities*^c^*	5	–	2
**Racial/ethnic identity**			
White	84	80	82
Biracial/Multiracial	5	11	2
Hispanic/Latinx	3	5	5
American Indian/Alaskan Native	3	3	2
Black/African American	0	0	4
Additional racial identities*^d^*	5	2	4
**Region of United States**			
Midwest	29	–	-
West	28	–	-
Northeast	27	–	-
South	16	–	-

*Not all numbers will total to 100, due to rounding.*

*^a^Nonbinary category includes the following labels: genderqueer, nonbinary, agender, genderless, two-spirited, gender non-conforming, gender fluid, androgynous, and trans feminine.*

*^b^No label category includes unknown, choose not to label, and “self-describe” option narratives, none of which labeled the person as binary female or male. For example, one read “Does not identify in any way yet.”*

*^c^Additional sexual identities include gay, questioning, and androsexual.*

*^d^Additional racial identities include Filipino, Asian Indian, Creole, and Native Hawaiian or other Pacific Islander, as well as one child with multiracial ancestry whose parent reported that they did not yet know how the child will identify.*

Most participants (87%) reported a social gender transition, a medical gender transition, or both, and 39% reported that this occurred after their eldest child was 18 months old. Participants reported having 1–2 children (*Mdn* = 1). Almost all children joined the family through biological means (98%), with just one child joining the family through adoption and none through foster care. Two-thirds of participants (*n* = 43) were biological parents to their eldest child (parent assigned female at birth *n* = 30; parent assigned male at birth *n* = 13).

### Procedure

We recruited participants between June 2016 and July 2017 through study advertisements posted on social media and online networking sites geared toward transgender parents (Tornello et al., 2019). The advertisement included participation criteria, a study description, and how to request a link to the survey. Participants could contact the PI (second author) via email or complete a study request form. If the participant was eligible to participate, they received a personalized link so that they and a partner (if applicable) could participate. Participants arrived first at a study consent page. If they agreed to participate, they were directed to the surveys. The Pennsylvania State University ethics review board (IRB) approved this study.

### Measures

#### Sex Assigned at Birth, Gender Identity, and Additional Demographic Information

Participants completed the two-question method for assessing sex and gender (Tate et al., 2014; Lagos and Compton, 2021) for themselves, their child, and their partner (if applicable). Participants first responded to this question: “What was [your/your partner’s/your child’s] sex assigned at birth (on [your/their] original birth certificate)?” Response options were *Male*, *Female*, and *Self-describe (please explain)* with an accompanying text box.

Participants then responded to this question: “What is [your/your partner’s/your child’s] current gender identity?” Response options were as follows: *Female, Male, Transgender female (MTF or FTF), Transgender male (FTM or MTM), Genderqueer, Gender non-conforming, Gender fluid, Nonbinary, Agender, Bigender, Choose not to label, Unknown, Two-spirited*, and *Self-describe (please explain)* with an accompanying text box. We coded whether parents labeled child gender identity dichotomously: *parent selected female or male response option* (1) or *parent selected “unknown” or “choose not to label” response option or wrote a narrative response that did not label the child as binary female or male* (0).

Participants also provided additional descriptive information about themselves, their children, and, if applicable, their partner (Tornello et al., 2019; see Table 1). We created a composite index (Gailmard, 2014) for SES by transforming education, participant hours worked, household income, and individual income into standardized *z* scores, then calculating participants’ mean *z* scores (Tornello et al., 2019). Higher *z* scores indicate higher SES (α = 0.77).

#### Child Gender Expression

The Pre-School Activities Inventory (PSAI; Golombok and Rust, 1993) is a 24-item measure used to assess gendered interests and behaviors in children aged 18–71 months. This measure lists masculinized and feminized toys (e.g., *toolset, jewelry*), activities (e.g., *climbing, playing house*), and personality characteristics (e.g., *enjoys rough and tumble play*, *likes pretty things*). Participants rate how often their child engages with or enjoys each item on a Likert scale from *never* (1) to *very often* (5). The PSAI was internally consistent (α = 0.73).

PSAI scores are calculated by summing responses to masculinized items, subtracting feminized items, standardizing the resulting scores, then adjusting for age norms (Golombok and Rust, 1993). Higher scores indicate more masculinized behaviors and interests and lower scores indicate more feminized behaviors and interests.

Standardized norms on the PSAI are distinct for male (*M* = 60, *SD* = 10) and female (*M* = 40, *SD* = 10) children. The PSAI manual (Golombok and Rust, 1993) does not specify whether these terms refer to sex assigned at birth or to current gender identity. No norms are available for children whose gender identity is not labeled or nonbinary.

#### Child Gender Expression: Conformity Versus Expansiveness

Following Farr et al. (2018), we transformed PSAI scores to calculate a variable, gender expansiveness, that allows comparisons across sex assigned at birth. We defined gender expansiveness as the extent to which a child’s PSAI score did not conform to societal expectations (Farr et al., 2018). During data collection, societal expectations for gendered behavior (from mass media, teachers, and peers, for example) were largely based on sex assigned at birth, as opposed to current gender identity. To create this gender expansiveness score, we subtracted age-adjusted PSAI scores (Golombok and Rust, 1993; Golombok et al., 2008) from the normed score for each child’s sex assigned at birth.

Negative scores indicate more gender conformity than typical among children in the normed sample. In contrast, positive scores indicate more gender expansiveness (a term we use here to indicate less conformity). A zero score indicates that the child’s gender expression conforms to gendered societal expectations based on a child’s sex assigned at birth.

#### Parent Gender Transition Timing

We assessed participants’ gender transition experiences (Tornello et al., 2019) by asking, “Have you transitioned in any way (socially, physically, psychologically, etc.)?” Response options were *Yes, No, Not applicable*, and *Self-describe (please specify).* Participants who responded *Yes* were asked, “When would you say you began your transition (estimate best you can)?” Response options were a text box labeled *MM/DD/YYYY* or *Self-describe (please explain).* We calculated a gender transition timing variable that indicated whether parents began transitioning after their eldest child reached 18 months old (when children begin to use gender labels; Martin and Ruble, 2010; coded as 1) or not (0). The latter category included parents who did not transition and those who transitioned before children reached 18 months old.

### Analytic Plan

We had four research questions. To address the first, we generated frequency tables for parents’ child gender identity labels and reported self-describe option themes.

Second, we compared child gender expression scores for children in the present study to those in the normed sample. Scores violated the assumption of homogeneity of variance, so we conducted Welch’s unequal variances *t*-tests. We then conducted an independent-samples *t*-test on data from the present study to compare children assigned female at birth to those assigned male.

Our third and fourth research questions predicted (Aim 3) labeling vs. declining to label a child’s gender identity and (Aim 4) children’s gender expression scores. We conducted multiple regressions with backward stepwise elimination for each question, using AIC to evaluate model performance. Initial models for both questions included all main effects and two-way interaction terms for child age, child sex assigned at birth, parent gender identity (nonbinary vs. binary), and SES; each model included one additional variable: child gender expression (Aim 3) and parent gender transition timing (Aim 4).

We conducted logistic regressions for Aim 3, as whether or not parents labeled child gender identity was dichotomously coded. In contrast, we conducted linear regressions for Aim 4, which has a continuous outcome. Parameters that increased the model AIC were included in the final model even if the parameter was not statistically significant (Konishi and Kitagawa, 2008); this occurred in Aim 3 analyses. These non-significant predictors were inadequately powered: main effect of child gender identity (observed power = 0.37), main effect of child gender expression (observed power = 0.05), and interaction between child age and child gender expression (observed power = 0.05).

## Results

### How Many Parents Labeled Child Gender Identity?

Most participants (59%, *n* = 38) reported a current gender identity for their child (cisgender boy *n* = 21, cisgender girl *n* = 16, transgender girl *n* = 1). Many participants (41%; *n* = 26) did not report their child’s current gender identity, choosing *unknown* (*n* = 15), *choose not to label* (*n* = 4), or *self-describe*, with a text field to write in their response (*n* = 6). These parents described either a gender-fluid child or a child who had not verbalized their gender identity. Several participants suggested that the child’s gender identity would become apparent over time, saying, “I think it’s too early to know” or “does not identify in any way yet.” Several participants described children who alternate between female and male identities or between binary and nonbinary identities or expression. One respondent reported that their child’s speech delay prevents the child from communicating a gender identity, so the parent doesn’t know the child’s gender identity. In contrast, all participants (100%) reported that their child was assigned a binary sex at birth.

### How Did Parents Describe Their Children’s Gendered Play?

**FIGURE 1:**
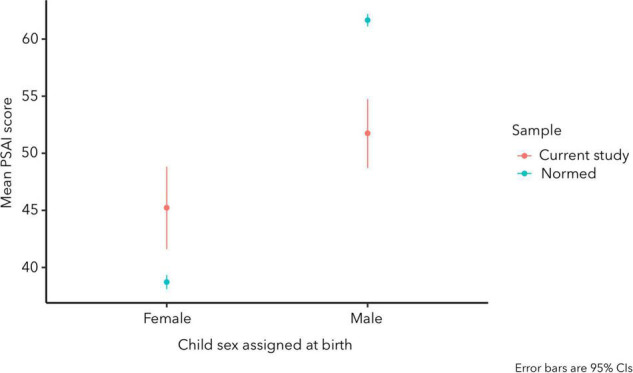
Pre-School Activities Inventory (PSAI) scores among children in the current study and in the normed sample as a function of sex assigned at birth, adjusted for child age.

We conducted Welch’s unequal variances *t*-tests to compare child gender expression scores for children in the present study to those in the normed sample (see Figure 1). Age-adjusted gender expression scores for children assigned female at birth in the present study (*M* = 45.23, 95% CI [41.60, 48.82], *n* = 25) were somewhat less feminized than for children assigned female at birth in the normed sample (*M* = 38.72; 95% CI [38.10, 39.34], *n* = 926; Golombok and Rust, 1993), *t*(25.50) = -3.57, *p* = 0.001. Similarly, gender expression scores for children assigned male at birth in the present study (*M* = 51.75, 95% CI [48.70, 54.75], *n* = 39) were less masculinized than for boys in the normed sample (*M* = 61.66, 95% CI [61.10, 62.20], *n* = 1166; Golombok and Rust, 1993), *t*(40.60) = 6.48, *p* < 0.001. In an independent-samples *t*-test comparing children in the present study by sex assigned at birth, gender expression scores for children assigned male were more masculinized than for children assigned female, *t*(53.00) = –2.78, *p* = 0.007, *d* = –0.70, 95% CI for *d* [–1.25, –0.19], as expected.

### Which Transgender and Nonbinary Parents Labeled Child Gender Identity?

The initial model predicting which parents labeled child gender identity (AIC = 85.06, *R*^2^ = 0.39) included all main effects and two-way interaction terms for child age, gender expression, sex assigned at birth, parent gender identity (nonbinary vs. binary), and SES. Backward stepwise elimination removed child sex assigned at birth, SES, and all but one interaction term from the final model, which accounted for almost a third of the variation in parental labeling (AIC = 70.74, *R*^2^ = 0.30; see Table 2).

**TABLE 2:** Final logistic regression model predicting nonbinary and binary transgender parents’ likelihood of labeling their child’s gender identity.

				95% Exp(*B*) CI			
						
Name of parameter^a^	Estimate	*SE*	Exp(*B*)	Lower	Upper	*z*	*P*
(Intercept)	0.78	0.36	2.18	1.12	4.63	2.18	**0.03**
Child age, in years	1.15	0.35	3.15	1.69	6.91	3.24	**0.001**
Child gender expression^b^	–0.05	0.03	0.95	0.89	1.01	–1.54	0.12
Parent gender identity^c^	1.09	0.69	2.98	0.79	12.43	1.58	0.11
Child age × gender expression	–0.04	0.03	0.96	0.91	1.01	–1.42	0.16

*Parents’ labeling of their child’s gender was coded as parent selected female or male response options for child gender (1) or parent selected “unknown” or “choose not to label” response option or described the child’s gender in narrative form without labeling the child as binary female or male (0).*

*^a^All variables were centered.*

*^b^As measured by the Pre-School Activities Inventory, adjusted for child age and centered around norms (Golombok and Rust, 1993) for the child’s sex assigned at birth (as in Farr et al., 2018). Lower scores correspond to greater gender conformity.*

*^c^Parent gender identity coded as 1 = binary transgender parent; 0 = nonbinary parent.*

*Bold values indicate p-values below 0.05.*

**FIGURE 2:**
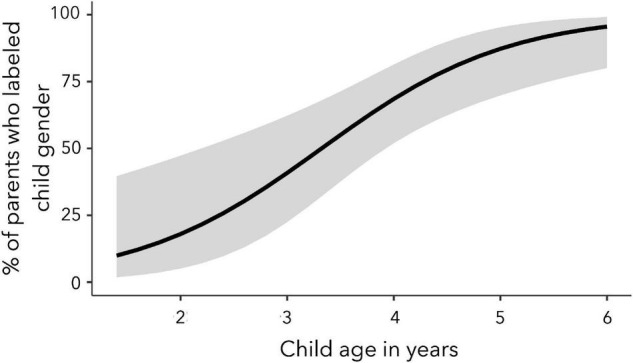
Child age was positively correlated with the likelihood that nonbinary and binary transgender parents would label their child’s gender identity.

The association between child age and child gender identity labeling likelihood was statistically significant, strong, and in the expected direction: the older the child, the more likely parents were to label their child’s gender identity (see Figure 2). Most (79%) binary transgender parents and roughly half (56%) of nonbinary parents labeled their child’s gender identity, and this difference was not statistically significant in the final model (*p* = 0.11). Parameters for the main effect of child gender expression (*p* = 0.11) and the interaction between child age and child gender expression (*p* = 0.13) were also not statistically significant in the final model.

### Which Transgender and Nonbinary Parents Described Their Children as Most Gender-Expansive?

The initial model predicting child gender expression (AIC = 358.36, *R*^2^ = 0.57) included all main effects and two-way interaction terms for child age, sex assigned at birth, parent SES, gender identity, and gender transition timing. Backward stepwise elimination removed parent SES, gender transition timing, and all but one interaction term from the final model. The final model accounted for almost half the variation in child gender expression (AIC = 343.84, *R*^2^ = 0.49; see Table 3).

**TABLE 3:** Final linear regression model predicting child gender expression scores^a^.

			95% CI			
					
Name of parameter^b^	Estimate	*SE*	Lower	Upper	*z*	*p*
(Intercept)	11.65	1.89	7.95	15.36	6.17	**<0.001**
Child sex assigned at birth^c^	–12.51	2.52	–17.46	–7.57	–4.96	**<0.001**
Parent gender identity^d^	–6.18	2.45	–10.99	–1.37	–2.52	**0.02**
Child age in years	5.27	1.42	2.48	8.06	3.70	**<0.001**
Parent gender identity × child age	–5.38	2.11	–9.51	–1.25	–2.55	**0.01**

*^a^As measured by the Pre-School Activities Inventory, adjusted for child age and centered around norms (Golombok and Rust, 1993) for the child’s sex assigned at birth (as in Farr et al., 2018). Lower scores correspond to greater gender conformity.*

*^b^All variables were centered.*

*^c^Child sex assigned at birth coded as 1 = male; 2 = female.*

*^d^Parent gender identity coded as 1 = binary transgender parent; 0 = nonbinary parent.*

*Bold values indicate p-values below 0.05.*

**FIGURE 3:**
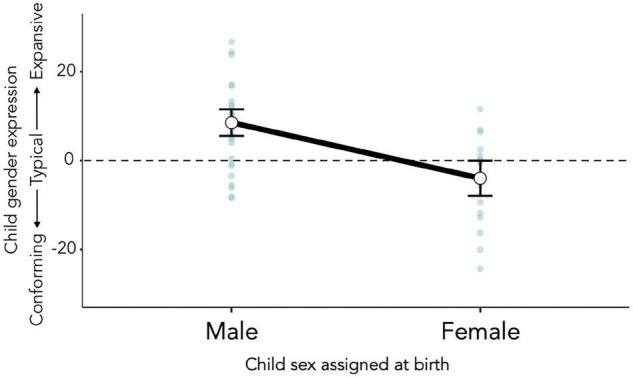
Gender expansiveness scores were significantly higher among children assigned male at birth than among those assigned female.

Child sex assigned at birth was the largest model contributor (Δ *R*^2^ = 0.29; see Figure 3). Gender expression scores indicated that children assigned male (estimated marginal *M* = 8.56, 95% CI [5.58, 11.55]) were more gender-expansive than those assigned female (estimated marginal *M* = –3.95, 95% CI [–7.88, –0.02]). An association between child age and gender expression was also statistically significant and in the expected direction: the older the child, the more expansive their gender expression scores.

As hypothesized, children with nonbinary parents had more gender-expansive scores (estimated marginal *M* = 5.40; 95% CI [1.82, 8.97]) than children in normed samples, as indicated by a 95% confidence interval that excludes zero. In contrast, children with binary transgender parents had scores similar to children in normed samples, as indicated by a 95% confidence interval that includes zero (estimated marginal *M* = –0.79; 95% CI [–4.09, 2.52]).

**FIGURE 4:**
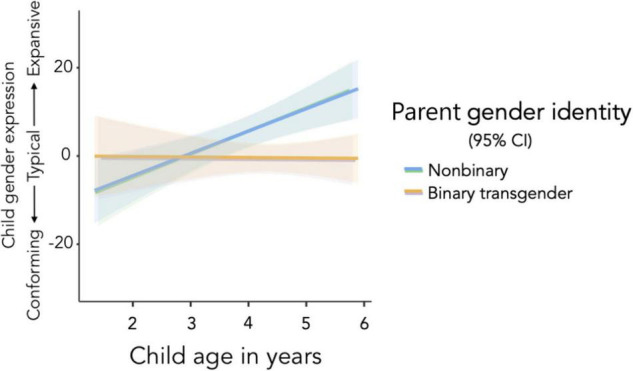
Child age was positively associated with child gender expansiveness among children with nonbinary parents, but these variables were not associated among children with binary transgender parents.

There was also a small and statistically significant interaction (Δ *R*^2^ = 0.07, see Figure 4). At all ages, children with binary transgender parents had estimated marginal mean gender expression scores that were not statistically different from zero. In other words, children had similar gender conformity levels across the 1.5- to 6-year age range whether they had binary transgender parents or participated in the normed sample. However, among children with nonbinary parents, mean-age (*M* = 4.06 years) and older children had estimated marginal mean scores significantly higher than zero. In other words, 4- to 6-year-old children with nonbinary parents had more expansive gender expression scores than same-age children in the normed samples. Among children with nonbinary parents, only the youngest age group (a standard deviation below the mean, corresponding to *M* = 2.85 years) had estimated marginal mean gender expression scores indicating typical gender conformity levels.

## Discussion

This study was the first to describe whether or not nonbinary and binary transgender parents label their child’s gender identity. As hypothesized, many nonbinary and binary transgender parents, particularly those with younger children, declined to label their child’s gender identity. Contrary to hypotheses, however, nonbinary and binary transgender parents were similarly likely to label their child’s gender.

This study also describes gender expression among children with nonbinary and binary transgender parents. There was some evidence for gender expansive patterns of play-based interests and behaviors. As hypothesized, transgender parents reported their children, particularly those assigned male at birth and older children with nonbinary parents, as engaging in more gender-expansive play than children in normed samples. However, just as in normed samples, there was also evidence for gendered patterns of play. Binary transgender parents described their children, across age, as playing in gendered ways, as did nonbinary parents with younger children. Overall, transgender parents described their children assigned female at birth as playing in more feminized ways than those assigned male.

Notably, effect sizes in this study were large, with final models accounting for much of the variation in parental labeling and child gender expression. We discuss each finding in turn.

Parents who decline to label their child’s gender identity on a survey may nonetheless assign their children gendered names, use gendered pronouns, and otherwise socialize them in gendered ways. However, our findings suggest that nonbinary and binary transgender parents may attend to the distinction between a person’s current gender identity and their sex assigned at birth (Blackless et al., 2000) and to children’s developing abilities to verbalize their gender identities (Fausto-Sterling, 2021). Notably, several participants stated that they were waiting for their child to verbalize a gender identity before labeling their child’s gender identity. The association between child age and parent labeling likelihood was strong, particularly when children were 2–4 years old, when children first label their own gender identity (Fausto-Sterling, 2021). Together, these qualitative responses and the quantitative pattern suggest that nonbinary and binary transgender parents may take a child-centered, developmentally aware approach, at least in some contexts. As transfamily theory (McGuire et al., 2016) suggests, they challenge common assumptions about gender in their families.

Transgender parents generally described their children’s play-based gender expression as more feminized among children assigned female at birth and as more masculinized among their children assigned male at birth. Participants reported, however, that their children, particularly those assigned male at birth, tended to play in more gender-expansive ways than children in normed samples. What are the likely mechanisms for such differences? Some evidence points to socialization influences: young boys adopted by lesbian mothers may also play in more gender-expansive ways than boys adopted by gay or heterosexual parents (Goldberg et al., 2012; Goldberg and Garcia, 2016). The gender-typed toy selection in the home can influence which toys children engage with and in turn, desire (Boe and Woods, 2018). Transgender parents may offer a “gender buffet” environment by offering children masculinized, feminized, and less gendered toys, which could cultivate greater gender expansiveness among these children (Averett, 2016). These findings suggest that lesbian and transgender parents may socialize their young children, in ways that lead to gender-expansive play.

Notably, nonbinary parents with older children described their children as more gender-expansive than did nonbinary parents with younger children, despite adjusting all scores for child age (Golombok and Rust, 1993). In contrast, child age was not associated with gender conformity among children with binary transgender parents. Together, this indicates that the association between child age and child gender conformity was stronger among children with nonbinary parents than among those with binary transgender parents (in the present sample) or cisgender parents (in the normed samples). A growing research body illustrates differences between nonbinary adults and binary transgender adults. Such differences include gender expression fluidity (Catalpa et al., 2019), gender binary challenging (Catalpa et al., 2019), gender identity self-awareness timing (Tatum et al., 2020; Puckett et al., 2021), and social expectation (that gender identity would or should be binary) awareness (Richards et al., 2016). This finding suggests that differences between nonbinary and binary transgender adults, such as generational differences between these two groups, may be linked with child gender expression (Tatum et al., 2020; Puckett et al., 2021).

Socialization that fosters gender expansiveness may benefit children as they develop. According to an American Psychological Association [APA] (2015) report, rigid masculinity concepts contribute to boys’ adverse outcomes, such as suppressing emotions in harmful ways. When parents’ gender ideologies are more flexible, their children express less pressure to conform to these gendered roles and expectations (Booth and Amato, 1994; Bos and Sandfort, 2010). Reducing this pressure may benefit children’s development overall (Blakemore et al., 2009; Martin et al., 2017), suggesting possible developmental advantages for children with nonbinary and binary transgender parents.

### Limitations, Future Directions, and Strengths

The current study has several limitations and strengths. The Gender Diverse Parents Study is based on a non-representative sample, and attempts to recruit transgender parents of color fell short, limiting generalizability. The Gender Diverse Parents Study subsample (parents of children under 6 years) was small. Participants were, however, diverse in geography, sexual identity, and SES. The key measure was the parent-report PSAI, and the validity of the PSAI has yet to be validated for use by transgender parents. Future research should investigate whether teacher reports or researcher observations would provide convergent validity for this measure in similar samples. PSAI guidelines and norms seem to be based on child sex assigned at birth (Golombok and Rust, 1993; Golombok et al., 2008; Farr et al., 2018), and there are no gender-identity-based PSAI norms for children without gender identity labels. However, the PSAI remains a psychometrically sound measure that has been validated in many contexts and has representative population norms. One current study strength is the analytic focus on within-group differences, which allowed for critical comparisons between families headed by nonbinary and binary transgender parents. This study is cross-sectional, so future researchers should address how within-family development may affect these findings over time. For example, school-age children with transgender parents are more likely than others to practice using affirming pronouns for their parents and educating their peers about gender identity (Zadeh et al., 2019).

Analysis of change over time was also not possible, given the design of the present study. Sociopolitical contexts affecting U.S. transgender people and their families have changed rapidly at national and local levels since data collection. At that time, gender identity was not a federally protected class, and identity labels that are now relatively outdated, such as MTF and FTM, remained common. During data collection, the primary source of oppressive anti-trans discourse was the 2016 U.S. presidential election (Pletta et al., 2022). As we write, state-level elected officials have become the primary oppressive anti-trans discourse sources. Future research is necessary to document the consequences of this state-level anti-LGBTQ legislation (Perez-Brumer et al., 2015; Du Bois et al., 2018) on children and parents alike.

Future research describing nonbinary and binary transgender parents’ and their children’s experiences is essential. Dunham and Olson (2016) have argued that multiracial, intersex, and transgender children can be essential participants in basic scientific research that has too often relied on discrete categories. We would add children with nonbinary and binary transgender parents to this list. Researchers should attempt replications in large, representative, and racially diverse samples with teacher or researcher gender expression observations, with enough statistical power to detect underpowered effects in the current study. Researchers should specifically design studies to understand how parent and child gender identity, race, ethnicity, SES, and sexual orientation intersect to influence child gender development. Future studies should explicitly measure parent gender expression, gender conceptualizations, and attitudes toward child gender expression to understand possible mechanisms better and distinguish between potential biological and socialization parental influence pathways.

## Implications and Conclusion

Many parents assume child gender based on sex assigned at birth, but we found that nonbinary and binary transgender parents may be less willing to label their child’s gender. As young children with transgender parents grow older and increasingly self-categorize their own gender identity, their parents may be increasingly likely to label their child’s gender. With age, these children (particularly those assigned male, those with nonbinary parents, or both) may also play in increasingly gender-expansive ways.

Future research should address how parent gender identity influences family processes, such as modeling of gender roles within the family, and by which mechanisms. Perhaps transgender parents actively or passively resist gendered peer, media, or co-parent influences so that children might experience less pressure to express their gender in more binary ways. Parental gender non-conformity modeling might empower children to express their gender expansively (Bruun and Farr, 2020). In contrast, some transgender parents may believe and may even encourage their children to exhibit *typical* gender expression, due to stigma against transgender parents (Rahilly, 2015).

Parents across gender identity who allow their children the freedom to explore their gender development may avoid potential misgendering harms (Tate et al., 2014) and provide them with supportive environments to explore their own gender identity and expression. Such expansive gender role expectations seem to benefit children (Witt, 1997; Blakemore et al., 2009; Martin et al., 2017; American Psychological Association [APA], 2018). These findings support transfamily theory (McGuire et al., 2016), with many nonbinary and binary transgender parents adopting a developmentally responsive, child-led approach to their children’s gender development. Researchers should further investigate links between parent gender identity, family processes, and child gender development.

## Data Availability Statement

The original contributions presented in this study are included in the article/supplementary material. Further inquiries can be directed to the corresponding author.

## Ethics Statement

This study was reviewed and approved by Pennsylvania State University. The participants provided their written informed consent to participate in this study.

## Author Contributions

RR designed the analytic plan, analyzed the data, and led the manuscript writing. ST designed and implemented the Gender Diverse Parents Study and co-wrote the manuscript. Both authors discussed the theoretical framework and results interpretation and contributed to the manuscript writing.

## Conflict of Interest

The authors declare that the research was conducted in the absence of any commercial or financial relationships that could be construed as a potential conflict of interest.

## Publisher’s Note

All claims expressed in this article are solely those of the authors and do not necessarily represent those of their affiliated organizations, or those of the publisher, the editors and the reviewers. Any product that may be evaluated in this article, or claim that may be made by its manufacturer, is not guaranteed or endorsed by the publisher.

## Footnotes

1 Roughly a third of transgender people identify as nonbinary (James et al., 2016; Carone et al., 2021), labeling their gender “outside the categories of female and male” (Hyde et al., 2019, p. 2). Many nonbinary people identify as genderqueer or genderfluid (Bradford and Catalpa, 2019).

2 Binary transgender people are those who (a) identify primarily as female and were assigned male at birth; or (b) identify primarily as male and were assigned female at birth (Bradford and Catalpa, 2019).

3 Transgender people (at least 0.4–0.5% of U.S. adults and 2% of U.S. youth; James et al., 2016; Meerwijk and Sevelius, 2017; Lagos and Compton, 2021) are people whose current gender identity is different from the sex they were assigned at birth.

4 Gender identity is a person’s internal sense of being, for example, a woman, man, nonbinary or genderqueer (Tate et al., 2014; American Psychological Association [APA], 2015).

5 Since inclusive gender identity measures are rarely used, nonbinary and binary transgender parents and children are unidentifiable in most gender development studies. Research has not yet addressed whether transgender or nonbinary children self-categorize their gender identity before the age of three years.

6 In contrast, many cultures across time and space have conceptualized gender identity as nonbinary, fluid over time, or not assumed based on sex assigned at birth (Mirandé, 2017).

7 Academic database searches for the terms “gender-creative parenting,” “gender-open parenting,” “theyby,” and “theybies” in August 2021 revealed no empirical studies about parents who avoided labeling their child’s gender identity based on the sex they were assigned at birth.

8 Some researchers have theorized that parental characteristics, such as parent gender identity or sexual orientation, might affect child gender identity (Green, 1978, 1998; Freedman et al., 2002; Goldberg et al., 2012; Fedewa et al., 2014), but evidence has not supported that conclusion. In existing studies, most children with transgender parents identified as cisgender themselves (Green, 1998; Freedman et al., 2002; Condat et al., 2020), like children with lesbian or gay parents (Goldberg et al., 2012; Fedewa et al., 2014) and children with cisgender single mothers (Stevens et al., 2002). McGuire et al. (2016) called for researchers to move beyond the cisnormative question “how many children with nonbinary and binary transgender parents are themselves nonbinary and binary transgender?” to better understand family processes in families headed by nonbinary and binary transgender parents.

9 Gender expression is a person’s external presentation of their gender through clothing, hairstyle, mannerisms, or other behaviors (American Psychological Association [APA], 2015).

10 Although most parents assign gender identity labels to their fetuses or newborns, assigning a child’s internal gender identity appears far less possible. Adults’ efforts to do so—either by attempting to “convert” nonbinary and binary transgender children or by arbitrarily assigning a gender label to an infant whose genitals were mutilated—are abusive and do not change the child’s internal gender identity (Diamond and Sigmundson, 1997; Tamar-Mattis, 2013; U.S. Substance Abuse and Mental Health Services Administration, 2015; Rafferty et al., 2018).

11 Child sex assigned at birth and age strongly predict children’s play-based gender expression: in population studies from the United Kingdom, United States, and Netherlands, boys and girls exhibit large differences, on average, in their gendered play interests and behaviors (Golombok and Rust, 1993; Golombok et al., 2008). Such differences are evident by 18 months and increase over the preschool years, with the most gender-conforming children becoming increasingly gender-conforming over time (Golombok and Rust, 1993; Golombok et al., 2008).
